# Effect of Plant Growth Regulators on Coloured Callus Formation and Accumulation of Azadirachtin, an Essential Biopesticide in *Azadirachta indica*

**DOI:** 10.3390/plants9030352

**Published:** 2020-03-11

**Authors:** Sharmilla Ashokhan, Rashidi Othman, Muhamad Hafiz Abd Rahim, Saiful Anuar Karsani, Jamilah Syafawati Yaacob

**Affiliations:** 1 Institute of Biological Sciences, Faculty of Science, University of Malaya, Kuala Lumpur 50603, Malaysia; sharmillaashokhan@gmail.com (S.A.); saiful72@um.edu.my (S.A.K.); 2 International Institute for Halal Research and Training (INHART), Herbarium Unit, Department of Landscape Architecture, Kulliyyah of Architecture and Environment Design, International Islamic University Malaysia, Kuala Lumpur 53100, Malaysia; rashidi@iium.edu.my; 3 Department of Food Science, Faculty of Food Science and Technology, Universiti Putra Malaysia, UPM Serdang 43400, Malaysia; 4 Centre for Research in Biotechnology for Agriculture (CEBAR), University of Malaya, Kuala Lumpur 50603, Malaysia

**Keywords:** neem, azadirachtin, coloured callus, thidiazuron, 2, 4-dichlorophenoxyacetic acid, biopesticide, sustainable agriculture

## Abstract

For centuries, *Azadirachta indica* or neem has been utilized as a primary source of medicine due to its antimicrobial, larvacidal, antimalarial and antifungal properties. Recently, its potential as an effective biopesticide has garnered attention, especially towards efficient and continuous production of its bioactive compounds. The present study investigated the effect of the plant growth regulators (PGRs) thiadiazuron (TDZ) and 2,4-dichlorophenoxyacetic acid (2,4-D) on the induction of colored callus formation and subsequent accumulation of azadirachtin (AZA) in *A. indica*. An efficient protocol was established for micropropagation and colored callus production of this species, followed by quantification of AZA (a mixture of azadirachtin A and B) and its safety assessment. For induction of the callus, leaf and petiole explants obtained from a young growing neem plant were excised and cultured on Murashige and Skoog (MS) medium supplemented with TDZ (0.2–0.6 mg L^−1^) and 2,4-D (0.2–0.6 mg L^−1^), either applied singly or in combination. Callus was successfully induced from both explant types at different rates, where media with 0.6 mg L^−1^ of TDZ resulted in the highest fresh weight (3.38 ± 0.08 g). In general, media with a single hormone (particularly TDZ) was more effective in producing a high mass of callus compared to combined PGRs. A culture duration of six weeks resulted in the production of green, brown and cream colored callus. The highest callus weight and accumulation of AZA was recorded in green callus (214.53 ± 33.63 mg g^−1^ dry weight (DW)) induced using TDZ. On the other hand, small amounts of AZA were detected in both brown and cream callus. Further experimentation indicated that the green callus with the highest AZA was found to be non-toxic (LC_50_ at 4606 µg mL^−1^) to the zebrafish animal model. These results suggested that the addition of different PGRs during in vitro culture could prominently affect callus and secondary metabolite production and can further be manipulated as a sustainable method for the production of a natural and environmentally friendly pesticide.

## 1. Introduction

Medicinal trees serve as one of the prominent sources of natural drugs, with about 80% of the world’s population still solely dependent on herbal or traditional medicine to treat various diseases [1,2,3]. Plant-derived pharmaceutical compounds are often preferred due to their safety, stability and affordability [4]. Among the widespread species of mahogany (Meliaceae), *Azadirachta indica* has been widely utilized in the healthcare and agriculture industries. Locally known as neem, this tree was mentioned in Ayurvedic medicine as one of the oldest remedies to treat various human diseases such as malaria, diabetes and skin infections, and it was also used as an appetite stimulator [5]. Neem extracts were also reported to possess inhibitory effects on several cancer cell lines such as breast, gastrointestinal, gynecological, hematological, prostate and skin cancers [6]. Based on literature surveys, over 300 structurally complex compounds have been identified [4,7]. Several classes of active chemical compounds had been discovered in neem such as nimbin, nimbidin, salanin, azadirachtin (AZA), polyphenolics, glycosides, dihydrochalcone, coumarin and tannins [8,9]. Nevertheless, the most predominant active compound in neem is AZA, which accounts for the majority of the biological activity of this species [10,11].

Neem has been conventionally propagated through seeds, however, there are many limitations caused by its recalcitrant properties and short-term viability [12]. There are also complications in selecting plants which are fast-growing and high-yielding in terms of secondary metabolite production and uniform in genetic constituents [13,14]. Commercialization of neem products are still in development due to insufficient natural resources and limited reports on its in vitro biochemical properties. To overcome these hurdles, tissue culture techniques can be applied as a sustainable alternative approach for mass and rapid propagation of this species, while minimizing the harvest of this plant from its natural habitat. Plant cell culture technology has served as an effective alternative system for the in vitro production of bioactive molecules to meet current market demands.

Besides that, modifications in terms of media composition and in vitro culture conditions are key elements in the production of targeted bioactive compounds. In the past few years, researchers have initiated callus lines from juvenile leaves and petioles of neem followed by the development of shoots and roots through the manipulation of growth regulators [15,16,17]. Indirect shoots and roots were also initiated from callus cultures of anther and leaf discs [18,19]. Furthermore, researchers have attempted to develop methods to produce valuable secondary metabolites in vitro. This technique serves as a valuable alternative to yield a continuous and sustainable production of secondary metabolites, as it is independent of climates and geographic location [20]. The yield of secondary metabolites produced through the conventional approach often varies depending on various other external factors such as soil composition, the presence of endophytic organisms, altitude, processing, and storage conditions [21,22]. On the other hand, the production of in vitro-derived secondary metabolites reduces the need for large land use and the heavy use of labor for planting, maintaining and harvesting the plants [23]. This method offers the potential for production scale-up through suspension culture and the use of bioreactors, genetically uniform starting material (clonal plants) as well as a more uniform secondary metabolite production, as all external factors affecting the growth of the plant materials can be controlled [24].

Preliminary studies are being conducted to elevate the continuous production of azadirachtin (AZA), a major compound which contributes to neem biological activities. Applications of AZA range from its utilization as an insect biocontrol agent, to vast medicinal and pharmacological applications. For example, AZA demonstrated larvicidal activity against *Anopheles stephensi*, a malarial vector [25], and possessed better antioxidant potential than ascorbate to scavenge free radicals [26]. Other than that, AZA was reported to possess chemo-preventive potential, whereby it inhibited the development of 7,12-dimethylbenz[a]anthracene (DMBA)-induced hamster buccal pouch (HBP) carcinomas, reportedly through the upregulation of antioxidant and carcinogen detoxification enzymes, reducing oxidative DNA damage and the prevention of pro-carcinogen activation [27]. Moreover, azadirachtin A has also been reported to exhibit hepatoprotective effects in rats, whereby treatments with azadirachtin A resulted in the amelioration of carbon tetrachloride (CCl4)-induced hepatotoxicity in a dose-dependent manner [28]. 

The first report on AZA production in androgenic haploid callus cultures was established by Srivastava and Chaturvedi [29], where anthers were used as explants to induce callus, followed by plantlet regeneration, which were then examined for AZA content. Akula et al. [30] have successfully induced somatic embryos from root and nodal segments, yet only a slight amount of AZA was detected in the derived cultures. Recently, AZA was also produced from transformed hairy roots of *A. indica*, which was cultured in a stirred tank reactor under optimized in vitro conditions and with the addition of elicitors [31]. Sujanya et al. [32] reported that nutritional variation influences the biomass content and AZA production.

Even though there are several reported publications on the production of AZA from neem in the literature, to date there is no available published report on the effect of 2, 4-dichlorophenoxyacetic acid (2, 4-D) and thidiazuron (TDZ) on the production of colored callus in *A. indica* and the subsequent assessment of their AZA (mixture of azadirachtin A and B) content. The present study was carried out to optimize and develop an efficient in vitro protocol for the production of callus of various colors in *A. indica* using different explants, as well as to quantify the levels of AZA in the colored callus and assess its toxicity. The outcomes of this study will yield valuable insights into the manipulation of culture conditions to produce colored materials (callus) with bioactive properties which can further be utilized as functional natural colorants or for the sustainable production of bioactive secondary metabolites such as AZA, a valuable biopesticide.

## 2. Materials and Methods

### 2.1. Explant Source and Surface Sterilization

Surface sterilization of explants was done according to Daud et al. [33] with some modifications. Leaf and petiole explants of a young and apparently healthy grown *A. indica* tree were collected. At first, the plant materials were washed under running tap water for an hour and then soaked in 0.3% (*w/v*) of Carbendazim (fungicide) solution for another hour. The traces of fungicide were rinsed off with distilled water and then soaked in 70% ethanol for 1 minute. The explants were rinsed with sterile distilled water once and soaked again in 70% (*v/v*) of commercially available bleach, Chlorox® (5.25% (*w/v*) sodium hypochlorite as the active ingredient), with the addition of a few drops of Tween 20 for 15 minutes. Finally, the traces of detergent were rinsed three times with sterile distilled water and inoculations of explants were done aseptically in a laminar flow hood.

### 2.2. Callus Cultures Establishment

Following surface sterilization, both leaf and petiole explants were blotted dry. Leaves were cut into pieces containing the midrib and two veins at each side of the midrib. Callus cultures were initiated on solid Murashige and Skoog (MS) [34] media comprising of 30 g L^−1^ sucrose, 4.4 g L^−1^ MS powder (Duchefa) and 8 g L^−1^ gelrite, supplemented with 2, 4-D (0.2 mg L^−1^, 0.4 mg L^−1^ or 0.6 mg L^−1^) and TDZ (0.2 mg L^−1^, 0.4 mg L^−1^ or 0.6 mg L^−1^) singly or in combination. Before autoclaving (at 121 °C for 20 min), the pH of the media was adjusted to 5.8. Explants were cultured in a sterile tube and were incubated in dark conditions in a growth room at 25 ± 1 °C. Visual observations of the morphology of the callus such as the color and texture, frequency of response, days taken for callus induction and fresh weight of the callus were recorded for six weeks.

### 2.3. Isolation and Extraction of Azadirachtin

On the sixth week of culture incubation, callus were harvested and grouped according to their color based on morphological observations (green/brown/cream). Samples were subjected to freeze-drying using a Labconco freeze dryer (Labconco Corporation, MO 64132 United States) at −50 °C. The extraction of AZA from the colored callus and preparation of the standard curve were done according to Singh and Chaturvedi [35] with some modifications.

Prior to extraction, colored callus were washed with distilled water and blotted dry. Thereafter, the samples were freeze dried until a constant weight was achieved. The drying temperature was kept low to prevent the thermal decomposition of metabolites. Prior to soaking, the samples were ground in a chilled mortar and pestle to enhance the extraction process. The whole extraction process was done in dim light conditions as the compounds are sensitive to light and heat. The dried samples (1–2 g DW) were soaked in methanol overnight at 4 °C, followed by sonification for 45 min. The mixtures were centrifuged using a refrigerated centrifuge at 5000 rpm for 10 min. The supernatant was collected and water was added at a ratio of 40:60 (40% water and 60% methanol). Following the addition of water, the mixture was partitioned against equal amounts of dichloromethane (DCM) in separating funnels. The solution was mixed thoroughly and was left to separate into two immiscible solvents (methanol + water and DCM). Following separation, the upper water–methanol layer was discarded and the lower phase of the DCM layer was collected and then evaporated to form dry matter. The retrieved dry matter was weighed and re-dissolved in HPLC grade methanol at a desired concentration and used as a stock solution. Then, the prepared extracts were filtered through a 0.22-mm nylon membrane filter prior to HPLC analysis.

### 2.4. HPLC Quantification of Azadirachtin 

Quantitation of AZA was conducted on an Agilent 1200 series HPLC system (Agilent Technologies, USA) comprised of a binary pump with an autosampler injector, micro vacuum degassers, a thermostatted column compartment and a diode array detector. A ZORBAX SB-C_18_ endcapped 5 µm, 4.6 mm × 250 mm reverse phase column (Agilent Technologies, USA) was used and the mobile phase was 90% methanol and 10% water at a flow rate of 0.5 mL^−1^. AZA was detected at 210 nm and the chromatographic peaks of the analytes were determined by comparing the retention time of the samples with an AZA standard (≈ 95%). The eluents used were as follows: (A) acetonitrile:water (9:1 *v/v*) and (B) ethyl acetate. The solvent gradient was developed by: 0–40% solvent B (0–20 min), 40–60% solvent B (20–25 min), 60–100% solvent B (25–25.1 min), 100% solvent B (25.1–35 min) and 100% solvent B (35–35.1 min) at a flow rate of 1.0 mL min^−1^. The column was allowed to re-equilibrate in 100% solvent A for 10 min prior to the next injection. The temperature of the column was maintained at 20 °C. The injection volume was 10 μL. The AZA compound was detected by co-chromatography with standards and by elucidation of their spectral characteristics using a photo-diode array detector. The quantity of the AZA compound was determined by comparing their relative measurement, as revealed by integrated HPLC peak areas. The overall AZA quantities in each colored callus were reported in terms of milligram per 1.0 dry weight of matter (mg/g DW).

### 2.5. Toxicity Analysis

#### 2.5.1. Sample Preparations and Zebrafish Maintenance and Breeding

For preparation of the extract for the zebrafish toxicity tests, the *A. indica* green callus was oven-dried at 33 °C, macerated and then soaked in methanol for 24 h (at 4 °C). Following that, the mixture was centrifuged at 5000 rpm for 10 min and the supernatant was collected and evaporated to dryness using a rotary evaporator at 50 °C. After that, the dried extract was reconstituted in Dimethyl sulfoxide, DMSO (1 g mL^−1^ of DW of green callus). For the purpose of the analysis, a stock solution (5 mg mL^−1^) of the green callus extract was prepared by dissolving the extract in sterile deionized water. The working solutions were prepared by diluting the stock solution in embryo media (Danio–SprintM solution) in 2-fold serial dilutions to six concentrations ranging from 500–5000 µg mL^−1^ in a 96-well microplate. Embryos in embryo media only (Danio–SprintM solution) were used as a negative control (untreated).

The keeping and raising of zebrafish (*Danio rerio* F. Hamilton, 1822) brood stocks were performed under the permission of the Institutional Animal Care and Use Committee (IACUC), Universiti Putra, Malaysia. Briefly, a pair of adult zebrafish was placed into a breeding tank prior to the day of breeding setup. On the following day, the embryos were collected using egg collector, washed and incubated in embryo media (Danio–SprintM solution) for approximately 2 hours. Dead (coagulated) embryos were discarded and only healthy fertilized embryos were selected for the zebrafish embryo toxicity assay.

#### 2.5.2. Zebrafish Embryo Toxicity (ZFET) Assay 

The zebrafish embryo toxicity assay was carried out based on the Organization for Economic Cooperation and Development (OECD) guidelines for fish embryo toxicity (FET) tests [36]. Briefly, 12 zebrafish embryos (one embryo/well) at 24 hours post-fertilization (24 hpf) were exposed to *A. indica* extract (200 µL) in 96-well microplates at six different concentrations ranging from 500–5000 µg mL^−1^. The toxicity of the extracts towards the zebrafish embryos was also compared to a commercial chemical pesticide (chlorpyrifos, 21.2% *w/w*) at the recommended field concentration (31.75 µg mL^−1^). Treated embryos were incubated at room temperature (25–28 °C) for 5 days. The cumulative mortality and developmental malformations of the embryos and larvae were observed and determined at every 24 h interval until 120 hours post exposure (hpe). Survival rate, hatching rate, heart rate, morphological malformation and teratogenic defects were observed and images/videos were captured and recorded using an inverted microscope attached to a digital camera. The heartbeat was counted from three selected embryos using a stopwatch for 1 minute. Lethal endpoints were characterized by coagulation and no heartbeat. Developmental anomalies such as pericardial edema, yolk sac edema, non-hatched, curved body and bent tail were recorded. The toxicity of the extracts was categorized according to Ohikhena et. al. [37], where plant extracts showing LC_50_ values greater than 1000 µg mL^−1^ are considered non-toxic, 500–1000 µg mL^−1^ are considered weakly toxic and less than 500 µg mL^−1^ are considered toxic.

### 2.6. Statistical Analysis

All tissue culture experiments were carried out in triplicate. Statistical analysis was done by one-way analysis of variance (ANOVA) followed by Duncan’s multiple range test (DMRT) at 5% significance level using SPSS software. All results were reported as mean ± standard error (SE) of triplicate experiments. For the zebrafish toxicity analysis, all graphs were generated by using GraphPad Prism version 7.0 (GraphPad Software, Inc.).

## 3. Results

### 3.1. Development of Callus in vitro

**Figure 1:**
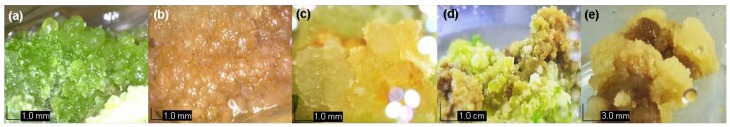
Production of colored callus from leaf explants of *Azadirachta indica*. (**a**) Green callus obtained from Murashige and Skoog (MS) media with added thiadiazuron (TDZ). (**b**) Brown callus obtained from MS media with added 2,4-dichlorophenoxyacetic acid (2,4-D). (**c**) Cream callus obtained from MS media with added 2, 4-D. (**d**) Green and brown callus obtained from MS media with added TDZ plus 2,4-D. (**e**) Brown and cream callus obtained from MS media with added 2,4-D.

In this study, the PGRs TDZ and 2,4-D were used either singly or in combination to induce the formation of callus of various colors in *A. indica* (Figure 1). The induction of callus formation from leaf explants cultured on single PGR treatments was observed after 14 days of culture on MS media with both 0.6 mg L^−1^ of TDZ and 2,4-D. Generally, the number of days taken for the leaf explants to respond reduced significantly as the concentrations of the PGRs used increased (Table 1). Interestingly, supplementation of only TDZ to the media yielded the formation of only green callus, in contrast to when 2, 4-D was added singly (Table 1). On the other hand, addition of only 2, 4-D to the media produced brown and cream callus (Table 1). Meanwhile, as shown in Table 2, the combination of both TDZ and 2,4-D in the media was observed to yield green callus at all concentrations used, but only produced brown callus when 2,4-D was combined with high TDZ concentrations (0.4–0.6 mg L^−1^). It was also observed that the initiation of callus formation from leaf explants was delayed when the hormones were used in combination.

In this study, the production of callus was also initiated from the petioles of *A. indica*. Our results showed that the initiation of callus formation from the petiole explants occurred faster when PGRs were used singly, producing callus as early as five days after culture (Table 3), but the response time was observed to increase when higher hormone concentrations were used. Similarly, petiole explants cultured on media only with added TDZ produced exclusively green callus (Table 3). However, the addition of 2,4-D to the petiole cultures was observed to yield both green and brown callus (Table 3).

Petiole explants were also cultured on media supplemented with both TDZ and 2,4-D (Table 4). Data analysis revealed that the combination of both TDZ and 2,4-D resulted in the formation of green callus at all PGR concentrations tested. In contrast, brown callus were only formed at high TDZ concentrations (0.4–0.6 mg L^−1^) and no formation of cream callus was seen from all single and combined PGR treatments (Table 3 and Table 4). Furthermore, the fresh weight of the callus produced from all treatments were also recorded to determine the most optimum callus induction media (CM) and the best explant type for callus production of this species. Overall, significantly higher callus biomass was obtained when leaves were used as the explant source (Table 1, Table 2, Table 3 and Table 4). Higher callus weights were also observed when the plant hormones were applied singly compared to when both TDZ and 2,4-D were used in combination (Table 1, Table 2, Table 3 and Table 4). TDZ supplementation also resulted in better callogenesis from the explants compared to 2,4-D, where MS media supplemented with only 0.6 mg L^−1^ TDZ was identified as the most optimum callus induction media (CM) for *A. indica*, yielding 3.38 ± 0.08 g of callus from leaf explants (Table 1).

### 3.2. Extraction and Quantification of Azadirachtin (AZA) from Colored Callus

**Figure 2:**
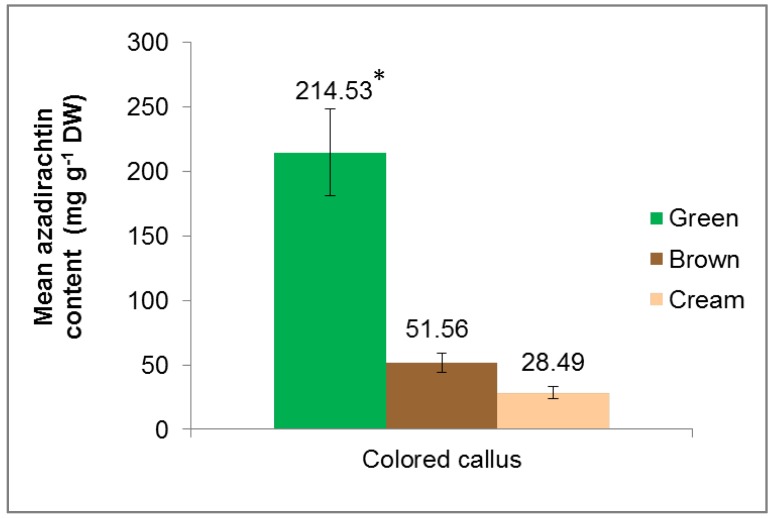
Mean azadirachtin (AZA) content (mg g^−1^ DW), from green, brown and cream colored callus.

The comparison of AZA content between green, brown and cream colored callus was carried out. Total AZA was extracted using absolute methanol, followed by liquid–liquid extraction using dichloromethane according to the protocol described by Singh and Chaturvedi [35]. We had previously reported that the colored callus of *A. indica* exhibits a wide range of biological actions such as radical scavenging and cytotoxic activities [38]. Due to these promising bioactivities, the colored callus obtained in this study (green, brown and cream) were evaluated for the presence of AZA, which is the most abundant bioactive compound of this species. The content of AZA in the colored callus extracts was quantified using HPLC (Figure 2). Based on the results, it was observed that green callus extract contained the highest level of AZA (214.53 ± 33.63 mg g^−1^ DW), followed by brown callus extract (51.56 ± 7.22 mg g^−1^ DW) and cream callus extract (28.49 ± 4.66 mg g^−1^ DW). These results are parallel to previously reported biochemical activities, which showed that green callus extracts possessed the highest bioactive compound content, antioxidant and cytotoxic potentials [38].

### 3.3. Toxicity Effects of Azadirachta indica Extracts on Zebrafish Embryos

**Figure 3:**
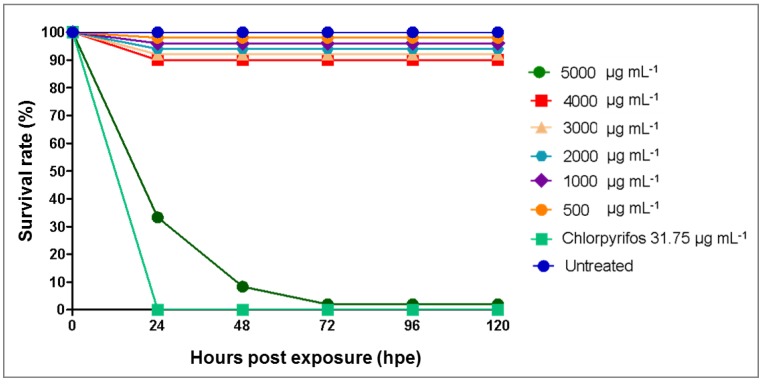
Survival rate of zebrafish embryos at 0 to 120 hours post exposure to *Azadirachta indica* extracts at concentrations ranging from 500–5000 µg mL^−1^ and commercial pesticide chlorpyrifos at 31.75 µg mL^−1^.

**Figure 4:**
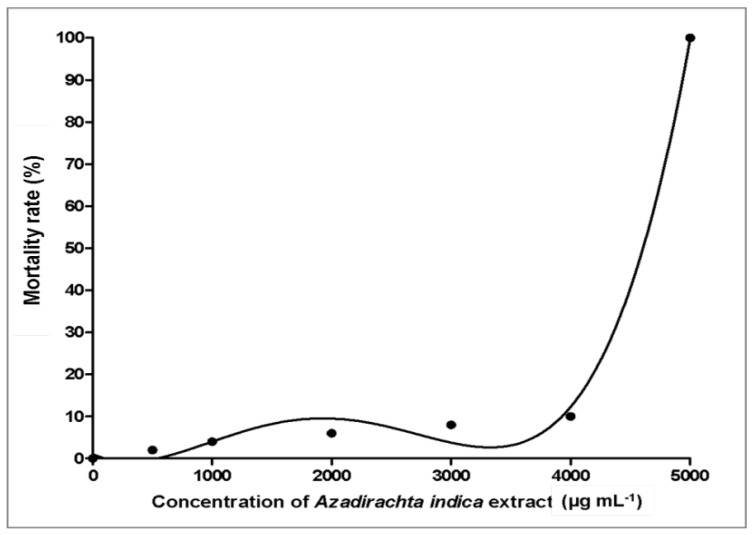
Effect of *Azadirachta indica* extracts (500–5000 µg mL^−1^) on the mortality rate of zebrafish embryos after 120 h post exposure (hpe). The median lethal concentration (LC_50_) value of *Azadirachta indica* extract resulting in 50% mortality of zebrafish embryos was 4606 µg mL^−1^.

*A. indica* extracts have been widely commercialized and used as a natural pesticide to protect plants from pest attacks. Thus, in this study, the possible toxicity of green callus extract was evaluated and compared to a common commercial chemical pesticide, chlorpyrifos, at its recommended field concentration (31.75 µg mL^−1^). Untreated embryos had a 100% survival rate throughout the analysis (Figure 3). Embryos treated with *A. indica* callus extracts at concentrations <4000 µg mL^−1^ had a high survival rate (90%), while embryos treated with 5000 µg mL^−1^ extract yielded a low survival rate (<10%) at 24 hpe which further declined to less than 5% after 72 hpe. In contrast, embryos exposed to chlorpyrifos at the recommended field concentration (31.75 µg mL^−1^) had a 0% survival rate after 24 hpe. Based on data analysis, the median lethal concentration (LC_50_ value) of the *A. indica* callus extracts was determined to be 4606 µg mL^−1^ (Figure 4), which was thus considered as non-toxic [37].

**Figure 5:**
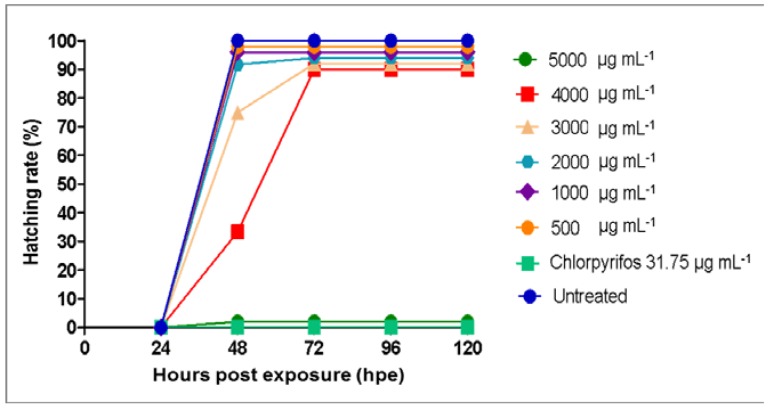
Hatching rate of zebrafish embryos after 0 to 120 hours post exposure with *Azadirachta indica* callus extracts at concentrations of 500–5000 µg mL^−1^.

**Figure 6:**
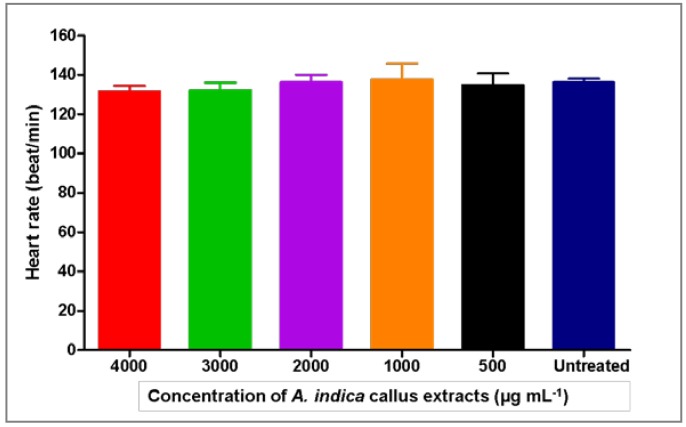
Effect of *Azadirachta indica* callus extracts at concentrations of 500–4000 µg mL^−1^ on heart rate of zebrafish larvae at 96 hours post exposure.

The hatching rate of zebrafish embryos upon treatment with *A. indica* callus extracts (500–5000 µg mL^−1^) was observed until 120 hpe (Figure 5). Results showed that the embryos treated with *A. indica* callus extracts at concentrations below 4000 µg mL^−1^ were able to hatch into larvae. In contrast, zebrafish embryos treated with 5000 µg mL^−1^ *A. indica* callus extract had a 0% hatching rate, similar to those treated with chlorpyrifos. Since the heart is the first organ to function during the development of many model organisms, including zebrafish [39], the heart rate of the resulting larvae was also monitored. Figure 6 depicts the heart rate of zebrafish larvae at 96 hpe after treatment with *A. indica* callus extracts, where the normal heart rate was recorded at 136 beats min^−1^. This data is in accordance with a previous report which indicated that the zebrafish normal embryonic heart rate is much closer to that of humans at 120–180 beats per minute [40]. Nevertheless, since *A. indica* callus extract at 5000 µg mL^−1^ resulted in a high mortality rate (>90%) at 96 hpe, the heart rates of zebrafish larvae at this concentration and those treated with chlorpyrifos solution were not determined.

**Figure 7:**
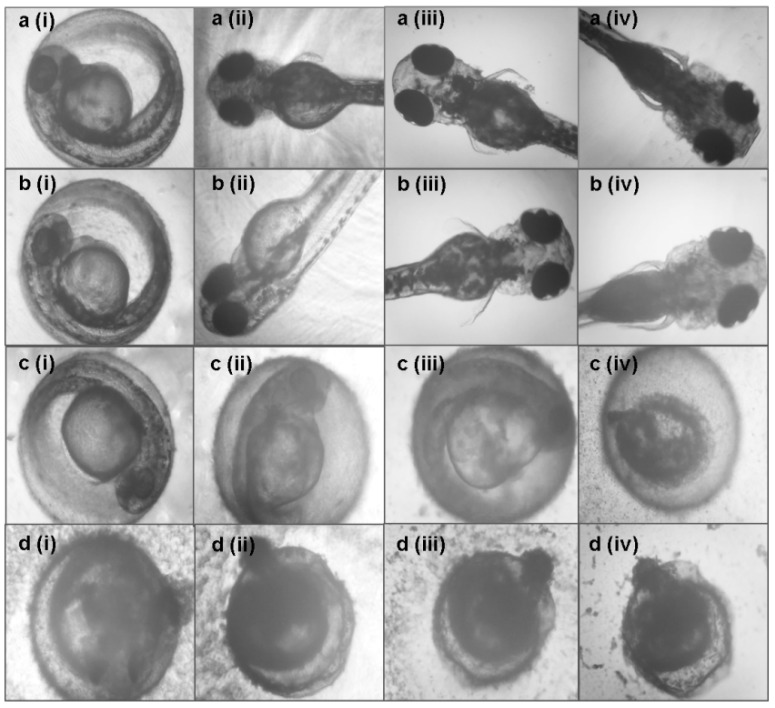
The effect of *A. indica* callus extracts and chlorpyrifos on zebrafish morphology. (**a**) Untreated; (**b**) 1000 µg mL^−1^ *A. indica* callus extract; (**c**) 5000 µg mL^−1^ *A. indica* callus extract; (**d**) 31.75 µg mL^−1^ chlorpyrifos at various time points: (**i**) 24 hpe, (**ii**) 48 hpe, (**iii**) 72 hpe, and (**iv**) 120 hpe. Images were captured using an inverted microscope at 100× (**i**) and 40× magnification (**ii**–**iv**).

Furthermore, the possible morphological defects of the embryos and larvae were also monitored and measured until 120 hpe. Results showed that there were no teratogenic defects observed in the embryos and larvae treated with *A. indica* callus extracts at all concentrations from 0 to 120 hpe (Figure 7). Exposure to *A. indica* callus extract at 5000 µg mL^−1^ inhibited the hatching of the embryos and killed the unhatched larvae (Figure 7c). On the other hand, treatment with 31.75 µg mL^−1^ chlorpyrifos caused coagulation after 24 hpe (Figure 7d).

## 4. Discussion

In this study, the leaf and petiole of *A. indica* were examined for their response when cultured on different types of plant growth regulators (TDZ and 2,4-D) at various concentrations, either singly or in combination. In most plant species, leaf explants have been shown to initiate the production of genetically identical cells by eliminating tissue culture-induced variability [41,42]. Plants naturally have the potential to facilitate alterations in their biological system towards any variations in their surrounding environments. Plant hormones and regulators are the key elements in the regulation of these environmental and developmental stimuli. Auxins and cytokinins are the most essential plant hormones as they are involved in regulating cell proliferation and differentiation which also explains the induction of meristematic activities. Exogenous PGRs such as cytokinins affect vascular differentiation and dedifferentiation in plant cells, which explains the formation of callus from the explants of *A. indica*. It has been reported that endogenous auxins such as Indole-3-acetic acid (IAA) start to accumulate at the petiole and basal internode once the explants are excised from the intact plant [43]. These explain the ability of both the leaf and petiole of *A. indica* to form dedifferentiated cells under in vitro conditions. Based on these results, leaf explants of *A. indica* had the optimum proliferation rate by producing 3.38 ± 0.08 g fresh weight of callus when supplemented with 0.6 mg L^−1^ TDZ, which was significantly higher compared to the callus biomass produced by petiole explants in all treatments. The varied callus induction ability between plant growth regulators can be explained by incompatible biochemical dissolution of bonds in the plant cell wall and different sensitivities towards cellular mechanisms [44]. This outcome was also supported by several studies which suggested young leaf explants are suitable for callus induction of most woody plants and shrubs such as *Litchi chinensis, Mesua ferrea, Alstonia scholaris*, *Tylophora indica* and *Digitalis mariana* [45,46,47,48]. Basically, leaf explants have the ability to adapt under in vitro conditions and attain competency to actively undergo mitotic divisions when supplemented with exogenous auxin and cytokinin [49,50]. However, it is worth noting that the use of growth media without PGR is preferred in tissue culture of endangered plants [51] as PGRs invoke callogenesis and the emergence of shoots from callus with a high incidence of hyperhydricity [52].

Even though several studies have been carried out on micropropagation of *A. indica*, very few studies have reported on the callus induction of this species in recent years. Our results showed that the application of TDZ singly at a concentration of 0.6 mg L^−1^ yielded optimum callus generation in *A. indica*. This is in contrast to a previously published report where 2,4-D was reported to cause optimum callus formation in *A. indica* either when applied singly or in combination with other plant growth hormones [13,53]. Nevertheless, very few studies have reported on the role of TDZ in the induction of callus formation in neem. So far, only one study has reported on the effect of combined TDZ and 2,4-D treatments on callus induction from neem leaves, although there are multiple reports on the in vitro effect of TDZ on other plants [54,55,56]. Akula et al. [30] reported that combinations of 2.3–4.5 mM TDZ and 0.5 mM 2,4-D yielded the highest formation levels of callus from neem leaves, in contrast to the findings of this study. The nature of TDZ hormone as substituted phenylurea promotes cell regeneration by its competency to accumulate endogenous cytokinin and facilitate the translocation of auxin, thus controlling the auxin/cytokinin ratio to promote callus growth. The rate of response is also solely dependent on the physiological state and metabolic rate of the explant [45,57]. In agreement with the outcomes of this study, several studies have reported that TDZ hormone tends to produce green and compact callus [58,59]. Meanwhile, similar to our results, supplementation of 2,4-D at various concentrations has been reported to induce the formation of soft and yellowish callus [55,56,60].

Although AZA has been recognized as an effective biodegradable pesticide [61], the current method of solvent extraction of this compound from the seeds of this species is unable to produce a sufficient economic yield of AZA. This is largely due to low variable yield, poor quality control, the presence of impurities and occurrence of diseases in the species [62]. Yield and purity improvement of AZA through conventional breeding have not produced positive results, mainly due to its recalcitrant properties. As a valuable alternative, plant tissue culture provides techniques for the possible mass production of in vitro neem cells, which can be utilized to mass-produce cell suspensions to serve as a channel for the substantial production of AZA. The current study provides the first report on the formation of callus of various colors (green, brown and cream) in neem, as well as the AZA content of the colored extracts. It was observed that green callus contained the highest amount of AZA (214.53 ± 33.63 mg g^−1^ DW) compared to brown and cream callus. The difference in AZA content among the colored callus extracts might be explained by various factors, such as the variation in organ differentiation type, totipotency, different levels of endogenous hormone activity or other cytoplasmic and physiological factors [59]. Several studies have suggested that the type and concentration of plant growth hormones could regulate the accumulation of bioactive compounds in plant tissue culture systems. For example, Grzegorczyk-Karolak et al. [63] mentioned that TDZ was most effective among other tested cytokinins in elevating secondary metabolite synthesis in shoot cultures of *Scutellaria altissima*. The significantly higher production of AZA in green callus (initiated by TDZ hormone application) highlights the potential of TDZ as a multi-purpose hormone to serve as an elicitor for secondary metabolite production in neem. 

Although the exact mechanism on how auxins such as 2,4-D influence the biosynthesis of secondary metabolites has not been fully understood, it had been reported to yield inhibitory effects on the production of anthocyanins [64]. This may explain why significantly lower levels of AZA were obtained in brown and cream callus produced through supplementation with 2 4-D. Nevertheless, the exact mechanism on how auxins influence the biosynthesis of secondary metabolites has not been fully understood. In addition, alterations in the carbon cycle of secondary metabolism, down-regulated key enzymes, irregular transport mechanisms and catabolism may also lead to the low production of AZA [65].

However, contrasting effects of combinatory PGR treatments on callus morphology and accumulation of AZA might be associated with various phenomena that took place during neem cell proliferation. Ontogenetic alterations that occur during the development of plant cells in artificial environments and defense mechanisms against added growth hormones are some known factors that influence the biosynthesis of compounds [66].

Although auxins such as 2,4-D have been reported to yield inhibitory effects on the production of secondary metabolites such as anthocyanins [64], the exact mechanism of how PGRs influence the biosynthesis of secondary metabolites has not been fully understood. Lower levels of AZA in brown and cream callus resulting from the addition of 2,4-D might be caused by the action of 2,4-D towards inhibiting the synthesis of secondary metabolites. Alterations in the carbon cycle of secondary metabolism, down-regulated key enzymes, irregular transport mechanisms and catabolism may lead to the low production of AZA [65].

Moreover, as pesticides are usually applied onto plants in farms through manual or automated spraying, the effects on their human handlers are often neglected. Also, if not used sparingly, pesticide runoff may flow into nearby lakes and rivers and affect aquatic animal inhabitants. Thus, in order to assess this, the toxicity of *A. indica* callus extracts were analyzed using zebrafish (*Danio rerio*) embryos. Zebrafish embryos/larvae were used as an animal model in this study as they offer several advantages and have been accepted as a reliable system to analyze any potential risks to human and ecological receptors [67,68]. The toxicity of the callus extracts was also compared to a commonly used chemical pesticide (chlorpyrifos at its recommended field concentration of 31.75 µg mL^−1^) to compare the safety of both on human handlers and other organisms. Data analysis revealed that *A. indica* callus extracts obtained in this study were not toxic, with an LC_50_ value of 4606 µg mL^−1^, while chlorpyrifos at 31.75 µg mL^−1^ may potentially cause harm to its human handlers and aquatic animals. Thus, it can be deduced that the green callus obtained in this study has the potential to be utilized for the production of a sustainable and environmentally friendly pesticide (recommended field concentration of AZA biopesticide is 95.39 µg mL^−1^). Meanwhile, human handlers working with chlorpyrifos are advised to wear proper attire and protective gear, and to use this pesticide sparingly.

## 5. Conclusions

The results of the present study show that different plant growth regulators (TDZ and 2,4-D) yielded differential results influencing the growth and development of callus in neem. When singly applied, 0.6 mg L^−1^ of TDZ has been determined as the most optimum callus induction media, producing 3.38 ± 0.08 g of green callus from leaf explants. Moreover, green callus were also observed to contain significantly higher amounts of azadirachtin (AZA) and its extracts were found to be non-toxic to zebrafish. Overall, this study delivers valuable insights into the potential improvisation of *A. indica* callus for the large scale production of biological materials containing valuable biochemical compounds (e.g., AZA) under controlled environments.

## Figures and Tables

**Table 1:** Effect of single TDZ and 2,4-D supplementation on the production of colored callus from leaf explants of *Azadirachta indica*.

MS + Hormone (mg L^−1^)	Fresh Weight (g) of Callus	Percentage (%) of Explants That Produced A Green Callus	Percentage (%) of Explants That Produced A Brown Callus	Percentage (%) of Explants That Produced A Cream Callus	Number of Days for Explants to Respond	Callus Texture
Control	0.22 ± 0.01 ^a^	NR	100.00 ± 0.00 ^a^	NR	41 ± 0.66 ^d^	watery
0.2 TDZ	2.08 ± 0.05 ^d^	100.00 ± 0.00 ^a^	NR	NR	20 ± 0.57 ^b^	compact
0.4 TDZ	2.41 ± 0.09 ^e^	100.00 ± 0.00 ^a^	NR	NR	15 ± 0.88 ^a^	compact
0.6 TDZ	3.38 ± 0.08 ^f^	100.00 ± 0.00 ^a^	NR	NR	14 ± 0.33 ^a^	compact
0.2 2,4-D	0.96 ± 0.06 ^c^	NR	100.00 ± 0.00 ^a^	60.00 ± 2.08	25 ± 0.33 ^c^	watery
0.4 2,4-D	0.82 ± 0.07 ^b,c^	NR	100.00 ± 0.00 ^a^	3.33 ± 1.52	16 ± 0.88 ^a^	watery
0.6 2,4-D	0.76 ± 0.03 ^b^	NR	100.00 ± 0.00 ^a^	16.67 ± 1.15	14 ± 0.33 ^a^	watery

Data represent mean value ± standard error (SE) with 30 explants in each treatment. Means with different letters in the same column are significantly different at *p* ≤ 0.05 according to Duncan’s multiple range test (DMRT). NR = no response.

**Table 2:** Effect of combined TDZ and 2,4-D supplementation on the production of colored callus from leaf explants of *Azadirachta indica*.

MS + Hormone (mg L^−1^)	Fresh Weight (g) of Callus	Percentage of (%) Explants That Produced A Green Callus	Percentage of (%) Explants That Produced A Brown Callus	Percentage of (%) Explants That Produced A Cream Callus	Number of Days for Explants to Respond	Callus Texture
Control	0.22 ± 0.01 ^a,b^	NR	100.00 ± 0.00 ^b^	NR	41 ± 0.66 ^e^	watery
0.2 T + 0.2 D	0.43 ± 0.04 ^d^	100.00 ± 0.00 ^a^	NR	NR	32 ± 1.15 ^d^	compact
0.2 T + 0.4 D	0.31 ± 0.03 ^c^	100.00 ± 0.00 ^a^	NR	NR	29 ± 0.58 ^c,d^	compact
0.2 T + 0.6 D	0.56 ± 0.03 ^e^	100.00 ± 0.00 ^a^	NR	NR	29 ± 0.66 ^c,d^	compact
0.4 T + 0.2 D	0.34 ± 0.04 ^c^	100.00 ± 0.00 ^a^	NR	NR	31 ± 0.57 ^d^	compact
0.4 T + 0.4 D	0.34 ± 0.03 ^c^	100.00 ± 0.00 ^a^	100.00 ± 0.00 ^b^	NR	31 ± 0.57 ^d^	watery + compact
0.4 T + 0.6 D	0.29 ± 0.03 ^b,c^	100.00 ± 0.00 ^a^	100.00 ± 0.00 ^b^	NR	31 ± 0.57 ^d^	watery + compact
0.6 T + 0.2 D	0.19 ± 0.03 ^a^	100.00 ± 0.00 ^a^	100.00 ± 0.00 ^b^	NR	18 ± 1.52 ^a^	watery + compact
0.6 T + 0.4 D	0.18 ± 0.02 ^a^	100.00 ± 0.00 ^a^	100.00 ± 0.00 ^b^	NR	23 ± 1.73 ^b^	watery + compact
0.6 T + 0.6 D	0.16 ± 0.02 ^a^	100.00 ± 0.00 ^a^	100.00 ± 0.00 ^b^	NR	27 ± 1.00 ^c^	watery + compact

Data represent mean value ± standard error (SE) with 30 explants in each treatment. Means with different letters in the same column are significantly different at *p* ≤ 0.05 according to Duncan’s multiple range test (DMRT). NR = no response; T = TDZ; D = 2,4-D.

**Table 3:** Effect of single TDZ and 2,4-D supplementation on the production of colored callus from petiole explants of *Azadirachta indica*.

MS + Hormone (mg L^−1^)	Fresh Weight (g) of Callus	Percentage of (%) Explants That Produced A Green Callus	Percentage of (%) Explants That Produced A Brown Callus	Percentage of (%) Explants That Produced A Cream Callus	Number of Days for Explants to Respond	Callus Texture
Control	NR	NR	NR	NR	NR	NR
0.2 TDZ	0.56 ± 0.17 ^d^	100 ± 0.00 ^a^	NR	NR	5 ± 1.52 ^a^	compact
0.4 TDZ	0.30 ± 0.08 ^a,b^	100 ± 0.00 ^a^	NR	NR	5 ± 0.67 ^a^	compact
0.6 TDZ	0.35 ± 0.12 ^b^	100 ± 0.00 ^a^	NR	NR	9 ± 1.54 ^c^	compact
0.2 2,4-D	0.57 ± 0.18 ^d^	100 ± 0.00 ^a^	100 ± 0.00 ^a^	NR	8 ± 0.53 ^b^	watery
0.4 2,4-D	0.34 ± 0.15 ^a^	100 ± 0.00 ^a^	100 ± 0.00 ^a^	NR	11 ± 1.66 ^e^	watery
0.6 2,4-D	0.42 ± 0.17 ^c^	100 ± 0.00 ^a^	100 ± 0.00 ^a^	NR	10 ± 0.75 ^d^	watery

Data represent mean value ± standard error (SE) with 30 explants in each treatment. Means with different letters in the same column are significantly different at *p* ≤ 0.05 according to Duncan’s multiple range test (DMRT). NR = no response.

**Table 4:** Effect of combined TDZ and 2,4-D supplementation on the production of colored callus from petiole explants of *Azadirachta indica*.

MS + Hormone (mg L^−1^)	Fresh Weight (g) of Callus	Percentage of (%) Explants That Produced A Green Callus	Percentage of (%) Explants That Produced A Brown Callus	Percentage of (%) Explants That Produced A Cream Callus	Number of Days for Explants to Respond	Callus Texture
Control	NR	NR	NR	NR	NR	NR
0.2 T + 0.2 D	0.20 ± 0.02 ^e^	100 ± 0.00 ^a^	100 ± 0.00 ^a^	NR	32 ± 1.15 ^b^	watery
0.2 T + 0.4 D	0.08 ± 0.00 ^d^	100 ± 0.00 ^a^	NR	NR	29 ± 1.15 ^b^	compact
0.2 T + 0.6 D	0.05 ± 0.00 ^c^	100 ± 0.00 ^a^	NR	NR	30 ± 0.58 ^b^	compact
0.4 T + 0.2 D	0.08 ± 0.01 ^d^	100 ± 0.00 ^a^	NR	NR	31 ± 0.58 ^b^	compact
0.4 T + 0.4 D	0.05 ± 0.00 ^c^	100 ± 0.00 ^a^	100 ± 0.00 ^a^	NR	31 ± 2.10 ^5^	watery + compact
0.4 T + 0.6 D	0.04 ± 0.00 ^b,c^	100 ± 0.00 ^a^	100 ± 0.00 ^a^	NR	31 ± 1.90 ^d^	watery + compact
0.6 T + 0.2 D	0.05 ± 0.00 ^c^	100 ± 0.00 ^a^	100 ± 0.00 ^b^	NR	18 ± 3.10 ^e^	watery + compact
0.6 T + 0.4 D	0.05 ± 0.00 ^c^	100 ± 0.00 ^a^	100 ± 0.00 ^b^	NR	23 ± 1.73 ^f^	watery + compact
0.6 T + 0.6 D	0.02 ± 0.02 ^b^	100 ± 0.00 ^a^	100 ± 0.00 ^b^	NR	27 ± 1.16 ^f^	watery + compact

Data represent mean value ± standard error (SE) with 30 explants in each treatment. Means with different letters in the same column are significantly different at *p* ≤ 0.05 according to Duncan’s multiple range test (DMRT). NR = no response; T = TDZ; D = 2,4-D.
